# 3D printed dielectric ceramic without a sintering stage

**DOI:** 10.1038/s41598-018-34408-5

**Published:** 2018-10-29

**Authors:** Maria Väätäjä, Hanna Kähäri, Katja Ohenoja, Maciej Sobocinski, Jari Juuti, Heli Jantunen

**Affiliations:** 1 Microelectronics Research Unit, P.O. Box 4500, 90014 University of Oulu, Oulu, Finland; 2 Fibre and Particle Engineering Research Unit, P.O. Box 4300, 90014 University of Oulu, Oulu, Finland

## Abstract

This paper presents for the first time the fabrication of dielectric ceramic parts by 3D printing without sintering. The printable paste was prepared by mixing a carefully selected amount of water-soluble Li_2_MoO_4_ powder with water. A viscous mixture of solid ceramic particles and saturated aqueous phase was formed with a solid content of 60.0 vol.%. Printing of the sample discs was conducted with material extrusion using a low-cost syringe-style 3D printer. The consolidation and densification of the printed parts occurred during both printing and drying of the paste due to extrusion pressure, capillary forces, and recrystallization of the dissolved Li_2_MoO_4_. Complete drying of the paste was ensured by heating at 120 °C. The microstructure showed no delamination of the printed layers. Relatively high densities and good dielectric properties were obtained, especially when considering that no sintering and only pressure from the extrusion was employed. This approach is expected to be feasible for similar ceramics and ceramic composites.

## Introduction

Fabrication of electroceramic parts by the conventional sintering method is both time- and energy-consuming. The process includes packing of the ceramic powder with organic additives under compression, followed by binder burnout and sintering at high temperatures. Sintering results in the densification of the powder into a solid piece due to thermally assisted mass transport^1,2^. The related shrinking is difficult to control, which respectively makes it challenging to control the size and shape of the final product. Consequently, additional shaping is often needed.

Additive manufacturing allows the fast production of parts even with complex shapes from polymers, metals, and ceramics without a requirement for special moulds or material-subtractive post-processing^3–5^. In this method, objects are made by adding material layerwise based on a virtual model. Techniques for this are various^4–6^. In general, ceramics need a secondary sintering step to densify the product. The only single-step technique for ceramics is the powder bed fusion method. This also utilizes thermal energy, but in the form of a laser, electron beam, or infrared lamps, to selectively fuse regions of a powder bed into a solid part^4,6,7^.

One relatively simple approach for additive manufacturing of structural and functional ceramics and thermoplastics^4,6^, is material extrusion, the selective dispensing of material through a nozzle either drop-wise or in a continuous manner^3,8^. Such methods that include deposition of material using a nozzle or a print head are also referred to as 3D printing^3^. Material extrusion is categorized as a direct method since the desired shape of the final part is achieved directly by depositing the material to its specific position^4^. The technique takes advantage of the liquid-to-solid state transition of the extruded material. During extrusion, the material is in the liquid state to enable flow under the applied extrusion pressure^9^. Materials used are either polymer melts or pastes containing a solid and a liquid phase with organic additives^4^. Subsequently, the deposited material solidifies to retain its shape and place thus facilitating the layer-by-layer manufacturing^9^. Depending on the material composition, the change to the solid state can be achieved by crystallization, liquid to glass transition, gelation, polymerization, dilatant transition, or solvent evaporation^9^. In general, the extrusion setup is relatively inexpensive^4^. It includes a build platform and a dispenser, either or both of them being movable, in addition to a nozzle^4,5^ which can easily be changed to have the required diameter (usually 100–1000 µm) depending on the desired precision and production rate^4^.

Lithium molybdate (Li_2_MoO_4_) is a non-toxic dielectric ceramic material, which has been studied for corrosion inhibition^10,11^ and moisture sensing applications^12,13^ as well as a scintillator material for detecting some rare nuclear processes^14,15^, anode material for Li-ion batteries in modified form^16,17^, and catalyst for methane oxidation^18^. For microwave devices, Li_2_MoO_4_ is of interest because of its beneficially low dielectric loss in addition to its low sintering temperature of 540 °C^19,20^. However, Li_2_MoO_4_ is water-soluble, enabling component manufacture at temperatures as low as room temperature^21–24^. In this method, referred to as room temperature fabrication (RTF), Li_2_MoO_4_ powder is moistened with water. Partial dissolution of the Li_2_MoO_4_ leads to formation of an aqueous phase which aids particle packing and densification during the compression. Thus, no shrinkage is observed. The dissolved Li_2_MoO_4_ recrystallizes during drying due to water evaporation, which can be speeded up by heat treatment at 120 °C^21^. The RTF method has also been applied in the manufacture of Li_2_MoO_4_ based composites to tailor the relative permittivity^22,23^ and its thermal coefficient^23^, or to introduce magnetic properties^25^. As no sintering is required, the unwanted formation of extra phases or heat expansion mismatch between the materials are not observed. Different types of antennas have also been manufactured successfully using RTF^24,26^.

Based on RTF, this paper presents a new approach to utilize Li_2_MoO_4_ and its water-solubility in the fabrication of dielectric ceramic parts by 3D printing. No organic additives or post-processing by sintering are required. It is the first attempt to fabricate 3D printed ceramic structures from a material that needs no sintering. For example, there is a need for complex 3D customized antenna structures^26,27^ and resonators with differing geometries^28–30^ which would benefit from the 3D printing. However, the proposed method to 3D print even simple shapes, as is done in this work, is useful in small-scale or prototype fabrication since it is mould-free thus providing flexible, low-cost design of the products before large-scale manufacturing.

## Results

### Paste properties

The printable paste realized in this paper resulted from the mixing of Li_2_MoO_4_ powder with a sufficient amount of water which partly dissolve the Li_2_MoO_4_. The composition of the paste was experimentally optimized to give a smooth uniform flow during the extrusion by keeping the amount of the water and the aqueous phase as low as possible. A low water content is desirable since the evaporation of water during drying results in residual porosity^23^. A smooth sample surface was obtained after printing.

**Figure 1:**
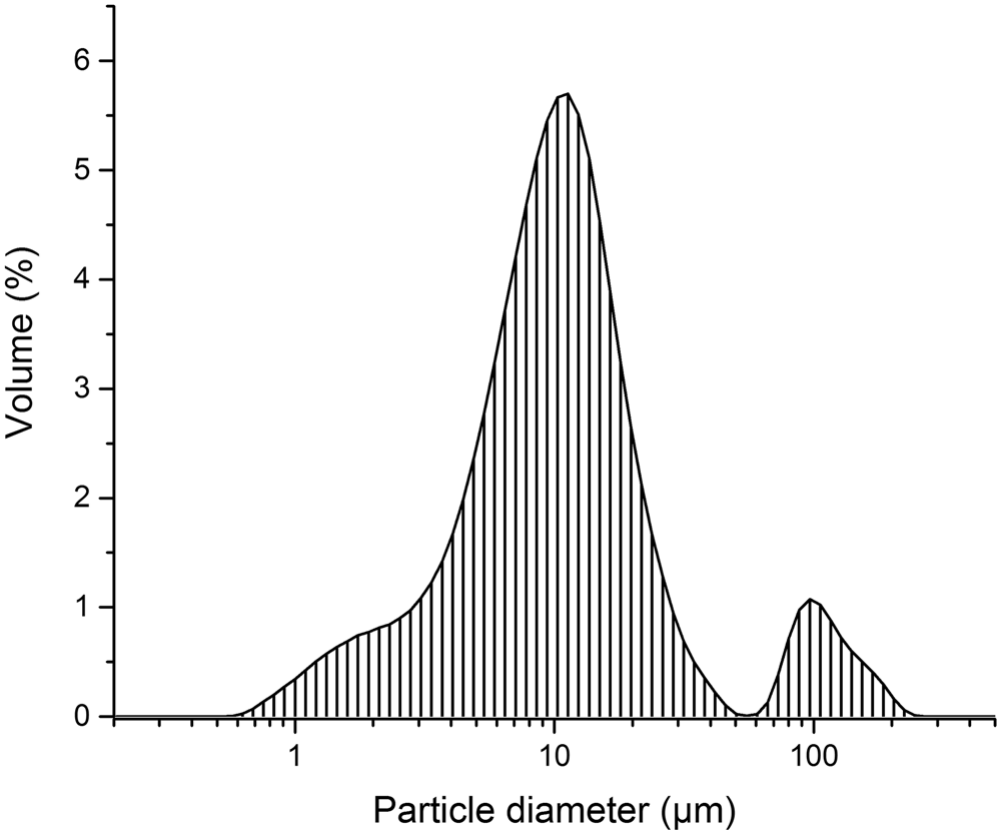
Particle size distribution of the Li_2_MoO_4_ powder showing the modes at around 10 and 100 µm.

**Figure 2:**
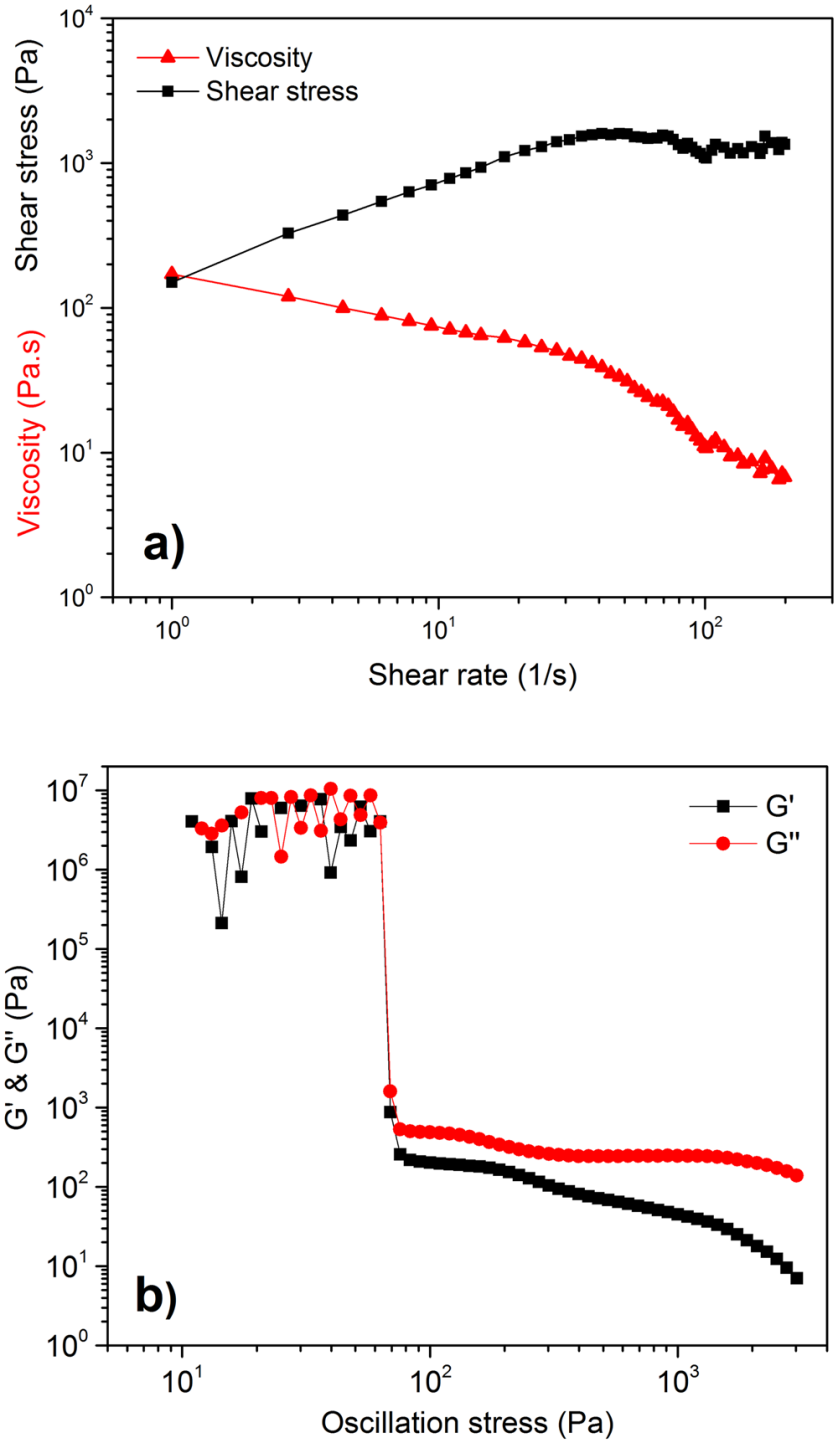
(**a**) Rheological behaviour of the paste showing shear-thinning behaviour, and (**b**) dynamic mechanical analysis at 1 Hz showing the yield point of 63.1 Pa.

Rheological properties, which determine the flow characteristics of the paste, are greatly affected by the particle size distribution of the solid powder as well as the by content of the liquid phase^2,31^. A large size difference between the particles improves the extrudability of the paste^32^. Fig. 1 presents the particle size distribution of the used Li_2_MoO_4_ powder. Modes at around 10 and 100 µm were observed with the mean particle size being ~20 µm. It should be noted, that as the Li_2_MoO_4_ partially dissolves upon mixing with water the size distribution of the solid particles in the paste or in the dry product is not equivalent to that shown in Fig. 1. The initial solid content was 67.6 vol.% mixed with 32.4 vol.% of water. Through a calculation with Li_2_MoO_4_ solubility (44.81 wt.% at 25 °C^33^, meaning 81.2 g per 100 mL of water) and density of Li_2_MoO_4_-water solution (1.4897 g/cm^3^)^34^, the solid content of the paste converted to 60.0 vol.% with 40.0 vol.% of saturated aqueous solution, respectively. Good flow properties under stress are required for successful extrusion^2^. The paste showed shear-thinning behaviour. (Fig. 2a). The yielding behaviour of the paste is shown in Fig. 2b. At low oscillation stress values the paste exhibited a linear viscoelastic response with some instability. The transition out of this region due to the disruption of the paste network could be clearly seen as both the G’ (storage modulus) and the G” (loss modulus) decreased. Using the transition point G’ = G”, the yield stress (called also the yield point) was 63.1 Pa.

### Microstructure

**Figure 3:**
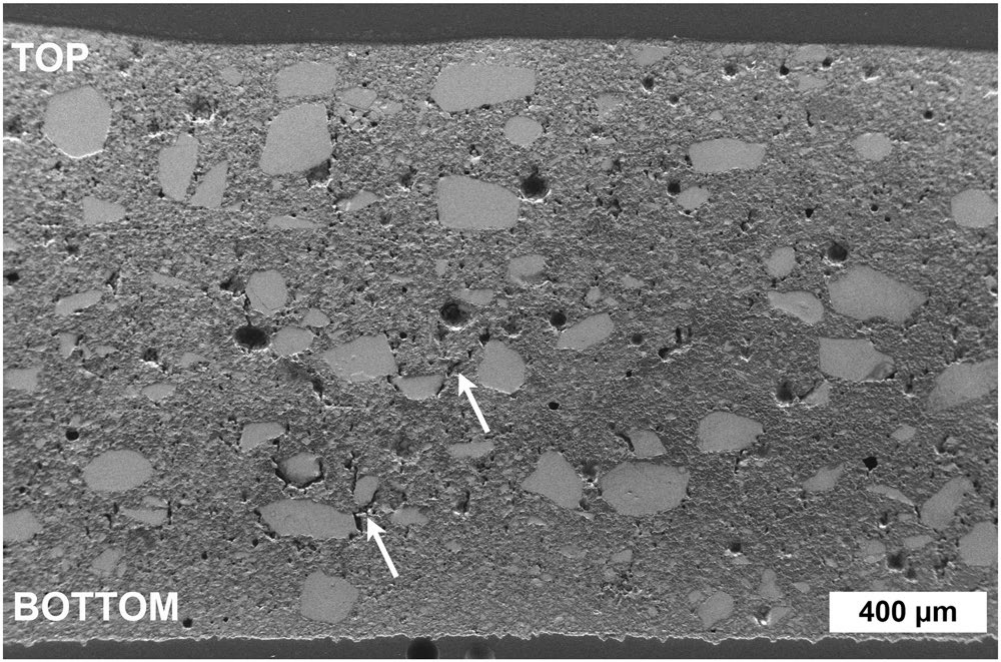
Secondary electron image of the dry sample cross-section along the printing direction shows the typical microstructure with bimodal particle size, pores in various sizes, and the densest microstructure in the vicinity of the top and bottom surfaces. The layer interfaces between the three layers are not observed. Minor cracks are indicated with arrows.

Cross-sectional analysis of the microstructure revealed the success of the printing and drying. A typical micrograph of a dried sample is shown in Fig. 3.

Microstructure showed similar bimodal particle size distribution as the original powder (Fig. 1). The larger particles (light grey colour) were embedded to the matrix of smaller ones (darker grey colour). Additionally, some spherical pores (dark spots) were observed with minor cracks (indicated with arrows) next to the larger particles. Interfaces between the three printed layers are not distinguished. Due to the densification mechanism, slightly denser microstructure could be observed in the vicinity of the top and bottom surfaces.

### Densities and dielectric properties

In addition to the microstructural analysis, the measurement of density also gave information on the porosity which has an impairing effect on the relative permittivity (ε_r_) and dielectric loss (tan δ). These factors determine the feasibility of the material in different electrical applications^35,36^. The measured densities and dielectric properties are reported in Table 1 in comparison with those reported for Li_2_MoO_4_ parts manufactured with RTF and post-processing at 120 °C^22^, and sintering at 540 °C^20,37^. It should be noted that the dielectric properties were measured from the samples thinned evenly from the bottom and top sides (thickness 0.87–0.94 mm) due to the requirements of the dielectric properties measurement device (see Methods). The densities shown in Table 1 are from the same samples. The densities of these thinned samples were naturally somewhat lower than those of the as-prepared samples with an original thickness of ~1.58–1.74 mm because the densest areas had been removed (Fig. 3).

**Table 1:** Densities and dielectric properties of Li_2_MoO_4_ ceramic samples fabricated with RTF based 3D printing, RTF with post-processing at 120 °C, and sintering at 540 °C.

	RTF based 3D Printing			RTF^22^	Sintering at 540 °C^20,37^
	Range of variation	Mean			
Densities					
Absolute (g/cm^3^)	2.31–2.44		2.40	2.83	2.895
Relative (%)	76–80		79	93	95.5
Dielectric properties					
Measurement frequency [GHz]	9.6			9.6	13.051
ε_r_	4.3–4.6		4.4	5.1	5.5
tan δ	0.0005–0.0006		0.0006	0.0004	0.00028

## Discussion

The amount of the liquid phase largely determines the rheology of the paste^31^. A high solid content increases viscosity due to the respectively high interaction frequency between the particles^38^. A sufficient amount of liquid is required to fill the interparticle voids and to overcome the friction between the particles by separating them from each other. The liquid phase also lubricates the movement of the paste along the dispenser during extrusion^31,39^. However, the size, size distribution, and shape of the solid particles affect the amount of liquid required^31^ and thus the optimal solid and liquid contents vary from case to case.

In general, successful printing depends on the printing rate and pressure. However, the extrusion system can provide only a limited amount of pressure which has to be taken into account when optimizing the rheological properties of the paste. For these low extrusion pressures, the flow resistance between the paste and the dispenser and nozzle walls should be low. Thus, the paste must have a low viscosity at the high shear rates related to these areas^31^. On the other hand, a high paste viscosity is required immediately after the printing as the shear stress is removed. This enables continuous extrusion of the paste which also must retain its position and shape after deposition with sufficient strength to bear the weight of the subsequent layers. At the same time, the paste still needs to fuse to the previously extruded layer for the final part to become uniform without delamination^8,31,38^.

For the paste to first flow and then set during and after printing, respectively, it should exhibit shear thinning behaviour i.e. a decreasing viscosity as a function of increasing shear rate^2,38,40^. This means that the apparent viscosity should be low enough at high shear rates to ensure that the paste is extruded smoothly through the nozzle without clogging^40–42^. In addition to this, the process of extrusion-based printing is always easier for materials with a low yield stress, i.e. the stress value below which the paste does not flow. This ensures the minimum pressure necessary to create the flow^43^. Also the paste has to have yielding property in order to maintain its shape after exiting the nozzle^38,40^. Setting of the paste can further be aided with a heated printing platform. This results in a decrease of the amount of liquid phase and an increase in the viscosity^38^.

In this study, the water content was kept as low as possible because an excess amount during the drying causes porosity^23^. Furthermore, keeping the liquid content low was beneficial because it shortens the drying time and reduces the related shrinkage and cracking^31,38,39^. Upon mixing, the liquid phase penetrated between the particles wetting them completely because of the Li_2_MoO_4_ solubility. The air between the particles now became located in the liquid phase as pores. The negative pressure in the pores, caused by the decrease in liquid-vapor surface area, provided a capillary force that rearranged the particles to give maximum packing, as reported earlier^44,45^. The paste was observed to exhibit beneficial shear thinning and yielding properties (Fig. 2). Previously, yield stress values of 83 and 30 Pa have been reported for aqueous alumina and mullite suspensions with organic additives and rheology modifiers having solid contents of 51 and 60 vol.%, respectively^46,47^. The measured yield stress in this study was 63.1 Pa with a solid content of 60.0 vol.%.

The driving force of the consolidation of the Li_2_MoO_4_ in the RTF method is pressure^21^ which in the case of extrusion depends on the paste rheology, the extrusion rate, and the dimensions and the geometry of the dispenser^31^. Immediately after deposition of the paste, consolidation continues with the aid of the liquid phase and related capillary forces. Evaporation of the water enhances the recrystallization of the dissolved Li_2_MoO_4_ which in this case was accelerated by a heated printing platform and post-processing at 60 and 120 °C. Due to consolidation by capillary forces after printing, shrinkage would be expected^45^. Accurate measurement of the shrinkage rate was impossible since immediately after printing the sample was too soft and after the drying step edges existed, which are typical for 3D printed parts^40^. However, the shrinkage was estimated at 3% when the diameters of the virtual model and the dried sample were compared.

The spherical porosity and minor cracks next to the larger particles observed in the microstructure (Fig. 3) may have originated from the coalescence of small pores into large ones due to capillary forces in the wet sample^45^. Large pores may occur as a result of possible air bubbles in the paste, accumulation of evaporating vapor inside a wet sample^45^, or due to the detachment of larger particles during polishing. Cracking can also be a sign of a too fast drying rate^40^. With further optimization of drying, for example in high humidity^40^, the porosity may be reduced but not totally removed as the evaporating water leaves some porosity behind^23^. However, the drying of the sample in this study was relatively successful because too fast a drying rate would also have resulted in warping and cracking^38,40^ and these defects were not observed (Fig. 3). The absence of layer interfaces implied good adhesion and fusion of the layers (Fig. 3). An earlier study^38^ suggests that upon optimal drying, the previously deposited layer has dried enough to induce fluid transport from the freshly printed layer. Fusion of the layers then occurs as some solid particles get dragged with the fluid^38^. However, in relation to the current case, the high density and viscosity of the saturated Li_2_MoO_4_ solution has been reported to impede mass transport^34^. Yet the densest microstructure observed in the vicinity of the top and bottom surfaces (Fig. 3) indicated liquid transport across the layer interfaces towards these drying surfaces. During drying, the liquid can diffuse from the interior part of the sample to the solid-air interface where the water then evaporates^48^. This results in recrystallization of dissolved Li_2_MoO_4_ in these areas and the formation of denser material compared to the interior part.

The measured densities (Table 1) were relatively high compared to the generally reported values (up to 60%) achieved in the ceramic parts after extrusion and drying^4^. However, the dielectric values were reasonable although the densities remained lower compared to those reported earlier for Li_2_MoO_4_ parts manufactured by simple pressing and drying (RTF)^22^ or pressing and sintering^20^. The theoretical relative permittivity of dielectric media with high porosity can be estimated by the Maxwell-Garnett equation^49^ (equation (1), see Methods). When permittivity values of 5.59 for Li_2_MoO_4_ (after porosity correction by equation (2), see Methods) and 1 for air were used, the resulting calculation gave theoretical permittivities of 4.4–4.7 which deviated only slightly from the measured values. The measured dielectric loss values had the same magnitude of (10^−4^) as those measured for samples manufactured with RTF^22^ and sintering^20^. However, the dielectric properties achieved make this fabrication method feasible for high frequency applications, especially if complex shapes are needed.

This work demonstrates for the first time the possibility of fabricating solid ceramic parts at room temperature directly by using 3D printing without the use of organic additives and sintering. With more detailed optimization of the paste rheology, printing parameters, and drying kinetics even further improvements of the microstructure should be achieved which in turn would have a beneficial impact on the dielectric properties. Microstructure also affects the mechanical properties and these still need to be determined. The results also indicate that other similar ceramics or ceramic composites could be fabricated with 3D printing. Moreover, the ability to manufacture ceramic parts without high temperature processing enables the direct and seamless integration of ceramics with temperature-sensitive materials such as polymers. The use of additive manufacturing technology by the printing of soluble ceramics and corresponding composites enables a vast number of applications, for example in electronics and telecommunication applications, and paves the way for significant time, cost, and energy savings compared to conventional ceramic processing.

## Methods

### Manufacturing and characterization of the paste

Li_2_MoO_4_ powder (99+ %, Alfa Aesar, Karlsruhe, Germany) was milled in ethanol with ZrO_2_ milling media in a planetary ball mill (Pulverisette 6, Fritsch, Idar-Oberstein, Germany) to reduce the particle size and to achieve a suitable size distribution. The powder was sieved with a mesh size of 200 µm and then analysed with a laser diffraction particle size analyser (Beckman Coulter LS13320, Brea, CA) in isopropanol. Ultrasonic mixing prior to analysis was omitted because it was observed to break down the larger particles. At least 99% of the particles were observed to be smaller than the chosen mesh size. To prepare the printing paste, 15.7–15.9 wt.% of deionized water was added to Li_2_MoO_4_ powder followed by a thorough mixing with a spatula and subsequent formation of a uniform viscous paste.

Rheological properties of the paste were studied with a rheometer (Discovery HR-1, TA Instruments, New Castle, DE) with Ø 40 mm parallel steel plate geometry and a 1 mm gap between the plates. A plastic cover was employed to prevent the paste from drying during the measurement. Prior to the measurements, the sample was conditioned to 22 °C and a pre-shear with 5 s^−1^ shear rate for 1 minute was applied followed by 2 minute equilibration period. Flow ramps were conducted at shear rates of 1–200 s^−1^. Dynamic mechanical analysis (DMA) was carried out at 1 Hz changing the oscillation stress from 0.1 Pa to 3000 Pa. The yield stress of the paste was determined by observing the stress at which the G’ is equal to the G”.

### 3D printing and post-processing of the samples

The disc-shaped samples (Ø ~ 25 mm, thickness ~1.5 mm) were printed with a material extrusion-type 3D printer (Leapfrog Creatr, Alphen aan den Rijn, Netherlands) equipped with a custom-made dispenser. Syringes having plastic tapered tips with a nozzle diameter of 0.84 mm (EFD Optimum, Nordson, Westlake, OH) were used.

Printing parameter settings with values empirically optimized for the current paste composition are presented in Table 2. Polyethylene terephthalate (PET) film was used as a substrate. A heated platform (80 °C), to which the substrate was attached, was employed to partially dry the paste after deposition from the nozzle in order to reduce its flow and to retain its shape as more layers were printed.

**Table 2:** Settings for printing parameters.

Parameter	Value
Printing height (with 0.3 mm offset)	0.80 mm
Printing width (printing line spacing)	0.60 mm
Extrusion rate (experimentally measured)	740 mm/min = 12.5 mm/s
Disperser movement speed	1000 mm/min = 16.7 mm/s

Prior to the printing of each sample, two exterior perimeters (Ø ~ 35 mm) were printed to ensure a proper flow of the paste. A total of three layers (thickness ~0.5 mm each) were printed per sample. Per layer, a perimeter was printed followed by a rectilinear infill. The infill angle was chosen to be parallel to that of the previous layer with suitable offsets (0.30 mm) in order to achieve the closest packed hexagonal layup to minimize the amount of space between the printed lines^40^.

After printing, the samples were left to dry on top of a mesh at room temperature for 66 h. After the first 18 h, the substrates were carefully removed to allow drying on both sides. Post-processing at 60 °C and 120 °C, for 24 and 6 h respectively, was employed to speed up the evaporation of the water and to ensure complete drying^22^.

Altogether, over 80 samples were printed using over 50 batches of paste, including the first trials and optimization of the printing and drying processes. 14 samples from 10 batches of paste were selected for characterization.

### Characterization of the samples

Measurement of the sample thicknesses was conducted with a micrometer screw (Mitutoyo Co., Kawasaki, Japan).

The microstructure of an as-prepared sample was characterized with a field emission scanning electron microscope using a secondary electron detector (Zeiss ULTRA Plus, Karlsruhe, Germany). The sample was mounted in epoxy for the imaging. The sample surface was polished using 1 µm water-free polycrystalline diamond suspension (Akasel, Cloeren Technology GmbH, Wegberg, Germany) and coated with a thin layer of carbon to eliminate electrostatic effects during imaging. Acquisition of the image was done with 2048 × 1536 resolution.

The high-frequency dielectric properties were measured with a non-contact method using a Split Post Dielectric Resonator (QWED, Warsaw, Poland), with a nominal resonant frequency of 9.97 GHz. Due to the sample height restriction of the device (h < 0.95 mm), the samples were accordingly thinned on both sides with a P1200 carborundum paper (EcoWet, KWH Mirka Ltd, Jeppo, Finland) and ethanol. The Maxwell-Garnett mixing equation

1 $${\varepsilon }_{eff}={\varepsilon }_{e}+3{V}_{f}{\varepsilon }_{e}\frac{{\varepsilon }_{i}-{\varepsilon }_{e}}{{\varepsilon }_{i}+2{e}_{e}-{V}_{f}({\varepsilon }_{i}-{\varepsilon }_{e})}$$

where *ε*_*eff*_ is the effective relative permittivity, *ε*_*e*_ permittivity of fully dense Li_2_MoO_4_, *ε*_*i*_ permittivity of air, and *V*_*f*_ volume fraction of air (porosity), was employed to estimate the theoretical permittivity^49^. The value for *ε*_*e*_ was calculated using the porosity correction equation

2 $${\varepsilon }_{e}={\varepsilon }_{meas}(1+1.5P)$$

where *ε*_*meas*_ is the measured relative permittivity, and *P* is the porosity^50,51^. For this calculation, the values used were *ε*_*meas*_ = 5.58 and *P* = 0.0437, as measured by Zhang *et al*.^52^ at 10.17 GHz frequency, which was close to the measurement frequency of the current work (Table 1).

Bulk densities were determined from the thinned samples to allow direct comparison with the associated dielectric properties. According to the standard for characterization of parts fabricated by additive manufacturing^53^, densities were determined with liquid impregnation using the vacuum method^54^. Due to the water-sensitivity of the samples, 1-butanol (≥99.4%, Sigma-Aldrich, St. Louis, MO) was used as an immersion liquid. 1-Butanol is non-toxic, has sufficiently low vapor pressure to be used with the vacuum, and it can be evaporated from the samples at 120 °C (the highest processing temperature of the samples) after measurements. To weigh the samples, Precisa ES 225SM-DR scale (Dietikon, Swizerland) was employed. The density of the 1-butanol (d = 0.808 g/cm^3^) at the prevailing temperature (21.3 °C) was measured using the scale equipped with a 350–8637 density determination kit (Precisa, Dietikon, Swizerland). The relative densities of the samples were calculated in relation to the theoretical Li_2_MoO_4_ density of 3.04 g/cm^3^ (Powder diffraction file 21–0763).

## Data Availability

All data generated of analysed during the current study are available from the corresponding author on reasonable request.
